# Potential of mean force between oppositely charged nanoparticles: A comprehensive comparison between Poisson–Boltzmann theory and Monte Carlo simulations

**DOI:** 10.1038/s41598-017-14636-x

**Published:** 2017-10-26

**Authors:** Jin-Si Zhang, Xi Zhang, Zhong-Liang Zhang, Zhi-Jie Tan

**Affiliations:** Center for Theoretical Physics and Key Laboratory of Artificial Micro & Nano-structures of Ministry of Education, School of Physics and Technology, Wuhan University, Wuhan, 430072 China

## Abstract

Ion-mediated interactions between like-charged polyelectrolytes have been paid much attention, and the Poisson–Boltzmann (PB) theory has been shown to fail in qualitatively predicting multivalent ion-mediated like-charge attraction. However, inadequate attention has been paid to the ion-mediated interactions between oppositely charged polyelectrolytes. In this work, the potentials of mean force (PMF) between oppositely charged nanoparticles in 1:1 and 2:2 salt solutions were investigated by Monte Carlo simulations and the PB theory. Our calculations show that the PMFs between oppositely charged nanoparticles are generally attractive in 1:1 and 2:2 salt solutions and that such attractive PMFs become weaker at higher 1:1 or 2:2 salt concentrations. The comprehensive comparisons show that the PB theory can quantitatively predict the PMFs between oppositely charged nanoparticles in 1:1 salt solutions, except for the slight deviation at very high 1:1 salt concentration. However, for 2:2 salt solutions, the PB theory generally overestimates the attractive PMF between oppositely charged nanoparticles, and this overestimation becomes more pronounced for nanoparticles with higher charge density and for higher 2:2 salt concentration. Our microscopic analyses suggest that the overestimation of the PB theory on the attractive PMFs for 2:2 salt solutions is attributed to the underestimation of divalent ions bound to nanoparticles.

## Introduction

Ions play essential roles in polyelectrolyte solutions and can modulate the effective interactions between polyelectrolytes^1–12^. In recent years, ion-mediated condensation/aggregation of like-charged polyelectrolytes such as nucleic acids and charged colloids have been investigated extensively by various experiments^1,13–21^. The existing experiments suggested that the Coulomb repulsion between like-charged polyelectrolytes can be weakened by the screening of monovalent ions and can be dramatically modulated into an effective attraction by the bound multivalent ions^13–21^.

For predicting bound ions to polyelectrolytes, some classical polyelectrolyte theories have been developed, such as the counterion condensation theory^22,23^, the Poisson–Boltzmann (PB) theory^24–32^, the density-functional theory^33^, and the tightly bound ion theory^34–41^. Among these theories, the PB theory has been widely used for polyelectrolyte systems and is rather successful in predicting electrostatic properties for polyelectrolytes such as colloids, nucleic acids, and proteins in aqueous/1:1 salt solutions^24–32,34–41^. However, because of the mean-field nature, the PB theory does not account for ion–ion correlations and consequently always predicts effective repulsions between like-charged polyelectrolytes even in multivalent salt solutions^42–45^. As an important bridge between theories and experiments, computer simulations including Monte Carlo (MC) simulations and molecular dynamics simulations have been proved to be effective tools in reproducing various experimental results and exploring the related microscopic mechanisms^46–53^.

Compared to systems of like-charged polyelectrolytes, less attention has been paid to systems of oppositely charged polyelectrolytes owing to the attractive nature of opposite-charge interactions. However, the aggregation or assembly of oppositely charged polyelectrolytes is a common phenomenon in biology, pharmacology, and material science^11,18,54–67^. For example, negatively charged DNA winds around positively charged histone to form nucleosome complex^18,54,55^. Another example is that, in gene therapy, DNA needs to be neutralized by complexation with polyelectrolytes or colloids with positive charges and so on^11,54,56^. In material science, oppositely charged colloids can be used to make controllable superlattices with potential photonic application^65–67^.

Importantly, the aggregation/assembly of oppositely charged polyelectrolytes is strongly dependent on the effective interaction between them. The existing experiments and theories including the PB theory and weak-/strong-coupling theory have shown that interactions between oppositely charged polyelectrolytes can be modulated by monovalent and multivalent salt ions^34,68–76^. In asymmetrical salt solution, repulsive interactions between oppositely charged polyelectrolytes were observed in experiments and simulations at very high salt concentrations^73–76^. Moreover, Borkovec *et al*. employed the PB theory with involving charge regulation to calculate the effective interactions between oppositely charged colloids in monovalent and multivalent ion solutions, and they compared the results with their experiments. They concluded that the PB theory can well describe the effective interactions between oppositely charged polyelectrolytes mediated by multivalent ions at large separation and low salt concentration^68–70^. The charge regulation involved in their PB calculations can be attributed to the change of effective charges on colloids due to ion binding or releasing in the inner layers of colloids^68–70^, and the charge regulation parameters were obtained through fitting their PB calculations to the experimental measurements for the corresponding symmetric colloid systems^68–70^. Therefore, it still remains unclear whether the PB theory in the original version can describe the effective interaction between oppositely charged polyelectrolytes, particularly in the presence of multivalent salt, given that the PB theory qualitatively fails to describe the multivalent-mediated attractions between like-charged polyelectrolytes^46,49,50^. In addition, there remains a lack of comprehensive understanding on the effective interaction between oppositely charged polyelectrolytes modulated by salt ions, especially multivalent salt ions.

In this work, we investigated the potential of mean force (PMF) between oppositely charged nanoparticles in symmetrical salt solutions by MC simulations and the PB theory. Our calculations were performed over wide ranges of ion conditions and charge densities on nanoparticles and would provide an overall illustration of ion-mediated PMFs between oppositely charged nanoparticles. In addition, the present work provides an extensive examination on when the PB theory works well for oppositely charged polyelectrolytes and microscopic analyses of the corresponding microscopic mechanism.

## Results and Discussion

**Figure 1:**
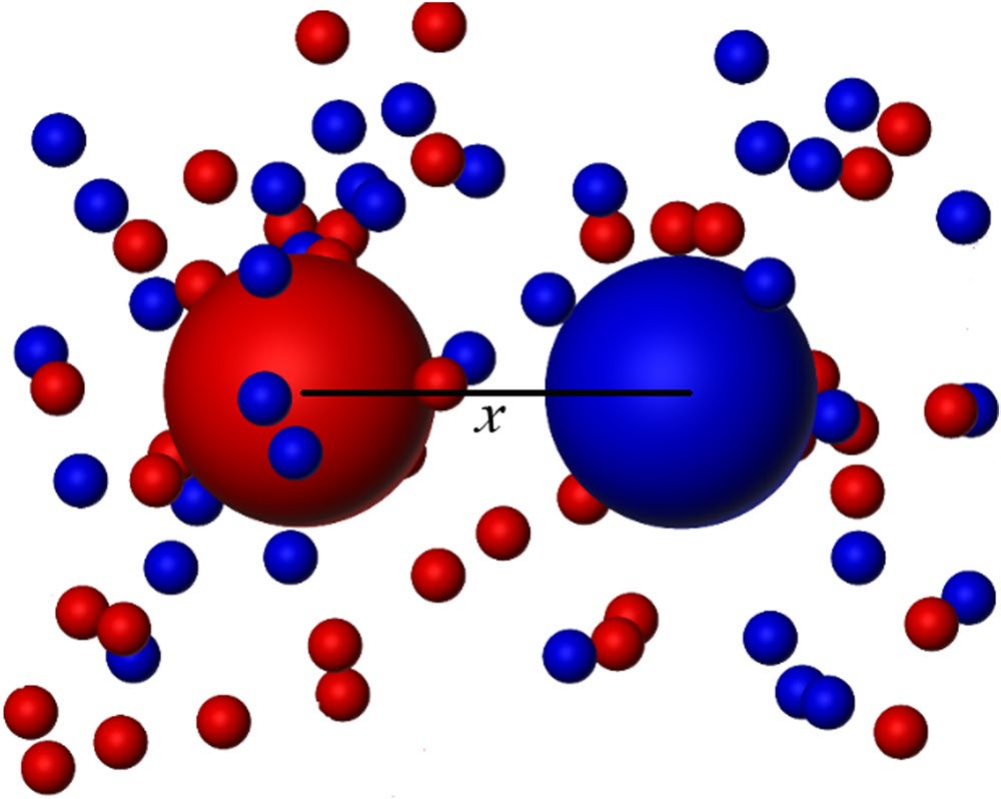
Illustration of two oppositely charged particles with a centre-to-centre separation *x* immersed in a *z*:−*z* salt solution. The large red and blue spheres represent positively and negatively charged nanoparticles, and the small red and blue spheres represent *z*-valent cations and −*z*-valent anions, respectively.

In this work, the PMFs *∆G*(*x*) between oppositely charged nanoparticles immersed in 1:1 and 2:2 salt solutions were calculated by the PB theory and MC simulations, as displayed in Fig. 1. Our calculations were performed over wide ranges of 1:1 and 2:2 salt concentrations and charge densities on nanoparticles. We have comprehensively compared the PB theory and MC simulations, and analysed when the PB theory works well and the corresponding microscopic mechanism in detail.

### 1:1 salt solutions

**Figure 2:**
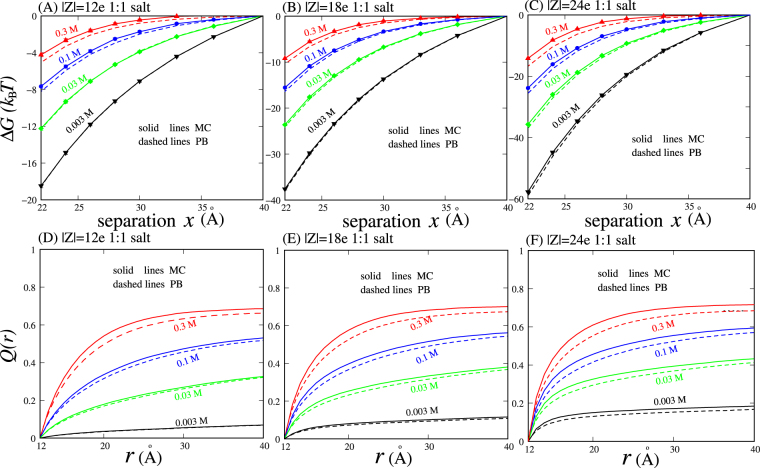
(**A**–**C**) Potentials of mean force Δ*G* as functions of separation *x* between the centres of two oppositely charged nanoparticles in 1:1 salt solutions, which were calculated by the PB theory and the MC simulations, respectively. (**D**–**F**) Net charge distributions *Q*(*r*) per unit charge on nanoparticles as functions of distance *r* around the nanoparticles with *x* = 22 Å in 1:1 salt solutions. The error bars are smaller than the symbols.

The PMFs between oppositely charged nanoparticles in 1:1 salt solutions were calculated over wide ranges of 1:1 salt concentrations and charge densities on nanoparticles. As shown in Fig. 2A–C and Fig. S1A,B in the Supplementary Material (SM), the PMFs between oppositely charged nanoparticles in 1:1 salt solutions are always attractive, and this attraction becomes weakened with increasing salt concentration. This is understandable because monovalent ions can bind to charged nanoparticles and reduce the opposite-charge Coulomb attraction, and higher ion concentration can cause stronger ionic screening for the oppositely charged nanoparticles owing to the lower entropy penalty for bound ions. Furthermore, with increasing charges on nanoparticles, the attractive PMFs become stronger. This is reasonable because the increasing charge density on nanoparticles can enhance the opposite-charge Coulomb attraction between nanoparticles and consequently enhance the ion-modulated attractive PMFs.

Figures 2(A–C) and S1A,B in the SM also show that the PMFs calculated from the PB theory are generally in quantitative accordance with those from the MC simulations for nanoparticles with different charges in 1:1 salt solutions, and the PB theory slightly overestimates the attractive PMFs between oppositely charged nanoparticles at very high 1:1 salt concentrations (e.g., ~0.3 M). This is not surprising even if the PB theory is generally considered to work well for polyelectrolytes in monovalent salt solutions. The PB theory assumes a continuous fluid-like ion distribution and consequently ignores the discrete properties of ions. For monovalent salt, although ion-ion Coulomb correlations are generally weak owing to the low valence, the exclusion volume correlation can become important at very high salt concentration^35,42–45,50^, and this exclusion correlation is ignored in the PB theory. As a result, the deviation between the PB theory and MC simulations for nanoparticles with *x* = 22 Å is approximately zero at low and moderate 1:1 salt concentrations, and it becomes visible at very high 1:1 salt concentration (e.g., the relative deviation of PMF ~10% at 0.3 M 1:1 salt), as shown in Figs S1 and S2 in the SM.

To gain a deep understanding on the PMFs, the bound ions around oppositely charged nanoparticles were analysed through calculating the net ion charge fraction *Q*(*r*) within a distance *r* from the centre of each nanoparticle. In our cases, the electrostatic potential is zero at the plane in the middle of two oppositely charged nanoparticles in symmetric 1:1 (or 2:2) salt, and this middle plane splits the space into two parts, in which the bound ions are dominated by the respective charged nanoparticles. Therefore, we calculate the net ion charge fraction *Q*(*r*) in one side of the middle plane^49,50,77^

1 $${Q}({r})=-\frac{1}{{Z}}{\int }_{ < r}{\sum }_{i}{z}_{i}{c}_{i}({\bf{r}}){d}^{3}{\bf{r}},$$

where Z is the charge on a nanoparticle, and *r* is a radial distance from the centre of a nanoparticle. *z*_*i*_ is the valence of ion species *i*, and *c*_*i*_(**r**) denotes its concentration at **r**. Here, the net ion charge fraction *Q*(*r*) represents the radial distribution of net bound ion charges and is generally calculated for the nanoparticles with separation *x* = 22 Å. The *Q*(*r*) values calculated by the PB theory and the MC simulations are shown in Figs 2D–F and S1C,D in the SM. For 1:1 salt, there is generally almost no difference in the net ion charge fraction *Q*(*r*) between the PB theory and the MC simulations, which corresponds to the quantitative agreement between the PMFs from the PB theory and the MC simulations. However, at very high 1:1 salt concentration (>0.1 M), the PB theory slightly underestimates the net ion charge fraction, suggesting fewer bound counterions to charged nanoparticles from the PB theory than those from the MC simulations. The slightly fewer bound counterions contribute to the above described slight overestimation on the attractive PMFs of the PB theory, because counterions provide the major contribution to reduce the Coulomb attraction between oppositely charged nanoparticles. Overall, the deviation between the predictions from the MC simulations and PB theory is insignificant on both the PMFs and the ion neutralization fraction for 1:1 salt solutions; see Figs S1 and S2 in the SM.

### 2:2 salt solutions

**Figure 3:**
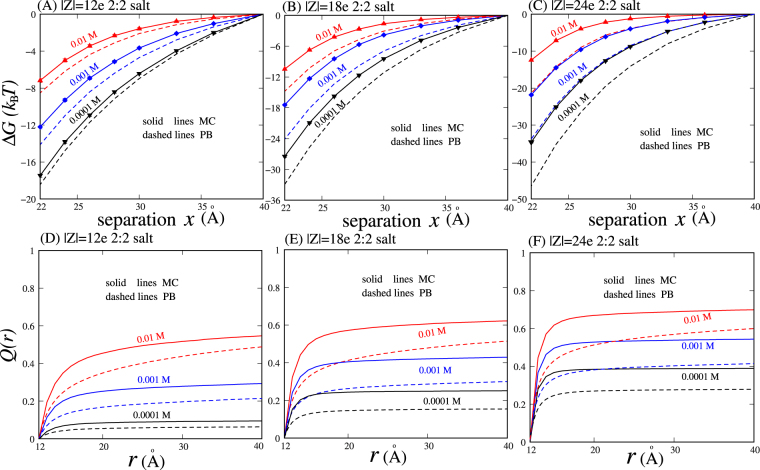
(**A**–**C**) Potentials of mean force Δ*G* as functions of separation *x* between the centres of two oppositely charged nanoparticles in 2:2 salt solutions, which were calculated by the PB theory and the MC simulations, respectively. (**D**–**F**) Net charge distributions *Q*(*r*) per unit charge on nanoparticles as functions of distance *r* around the nanoparticles with *x* = 22 Å in 2:2 salt solutions. The error bars are smaller than the symbols.

The PMFs between oppositely charged nanoparticles in 2:2 salt solutions were calculated over wide ranges of 2:2 salt concentrations and charge densities on nanoparticles. As shown in Figs 3A–C and S3A,B in the SM, in analogy to 1:1 salt, the PMFs between oppositely charged nanoparticles are always attractive in 2:2 salt solutions. This effective attraction becomes weakened with increasing 2:2 salt concentration, which is attributed to more bound ions at higher ion concentration.

Figures 3A–C and S3A,B in the SM show that compared with the PMFs from the MC simulations, the PB theory generally overestimates the effective attraction between oppositely charged nanoparticles in 2:2 salt solutions. This overestimation becomes more pronounced for nanoparticles with high charge densities and for high 2:2 salt concentrations. For example, for the nanoparticles with |Z| = 12e (e is the elementary charge) in 0.1 mM 2:2 salt solution, the relative deviation of PMFs at *x* = 22 Å between the PB theory and MC simulations is ~7% and can become as large as ~73% for nanoparticles with |Z| = 24e in 10 mM 2:2 salt solution. This suggests that for 2:2 salt, the PB theory generally qualitatively predicts the effective interactions between oppositely charged nanoparticles rather than quantitative predictions, and the deviation of the PB predictions can become very pronounced for nanoparticles with high charges in high 2:2 salt solutions.

To understand the severe deviations between the PMFs from the PB theory and MC simulations, we analysed the bound ions around oppositely charged nanoparticles by calculating the net ion charge fraction *Q*(*r*) within a distance *r* from the centre of each nanoparticle; see Eq. 1. As shown in Figs 3D–F and S3C,D in the SM, the net ion charge fraction *Q*(*r*) from the MC simulations is generally greater than that from the PB theory except for the nanoparticles with very low charge density. The underestimation of the PB theory on *Q*(*r*) becomes more pronounced for nanoparticles with higher charge density and for higher 2:2 salt concentration. For example, ∆*Q*(*r*) = (|*Q*_MC_(*r*) − *Q*_PB_(*r*)|) can become as large as ~0.25 at *r* = 14 Å for |Z| = 24e and 10 mM 2:2 salt concentration. The underestimation of the PB theory on *Q*(*r*) is in accordance with the above described overestimation on the attractive PMFs between oppositely charged nanoparticles in 2:2 salt solutions. The apparent underestimation of the PB theory on bound ions is attributed to the high charge of 2:2 salt ions, which can cause strong Coulomb correlations between divalent ions. As described above and previously^35,40,42–45,50,78,79^, the PB theory assumes fluid-like continuous ion distributions and ignores the correlations between ions, whereas the MC simulations explicitly account for the ion-ion correlations. The ion-ion correlations can drive ions to self-organize to low-energy micro-states and generally cause more bound ions to a charged polyelectrolyte^35,40,42–45,50,78,79^. Therefore, the PB theory generally underestimates the bound divalent ions and consequently overestimates the attractive PMFs between oppositely charged nanoparticles in 2:2 salt solutions. With increasing charge density on nanoparticles or increasing 2:2 salt concentration, the ion-ion correlations near the surface of nanoparticles would become stronger, and consequently the deviation of the PB predictions on bound ions and the resultant PMFs would become more pronounced; see Figs S3 and S4 in the SM.

### 1:1 salt versus 2:2 salt

Physically, multivalent ions can interact more strongly with charged nanoparticles and consequently can bind more strongly to charged nanoparticles than monovalent ions. The larger *Q*(*r*) of bound multivalent ions relative to monovalent ions becomes more pronounced for nanoparticles with higher charges; see Figs 2D–F and 3D–F. As a result, compared with 1:1 salt, 2:2 salt is more efficient in screening the Coulomb attraction between oppositely charged nanoparticles, and this efficiency becomes more pronounced for nanoparticles with higher |Z|, as shown in Figs 2 and 3. For example, in 30 mM 1:1 salt and 1 mM 2:2 salt solutions, the attractive PMFs are almost identical for the nanoparticles with |Z| = 12e, whereas for the nanoparticles with |Z| = 18e, the PMF in 1 mM 2:2 salt solution becomes visibly less attractive than that in 30 mM 1:1 salt solution.

**Figure 4:**
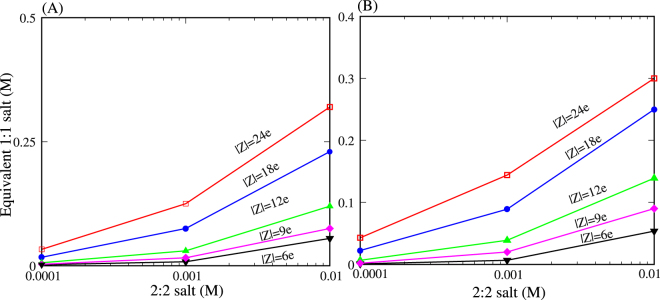
(**A**) Equivalent 1:1 salt concentration as a function of 2:2 salt concentration. Here, a 2:2 salt concentration and the corresponding equivalent 1:1 salt concentration can modulate the (nearly) same attractive PMFs ∆*G* (*x* = 22 Å) between oppositely charged nanoparticles. (**B**) Equivalent 1:1 salt concentration as a function of 2:2 salt concentration. Here, a 2:2 salt concentration and the corresponding equivalent 1:1 salt concentration can cause the (nearly) same *Q*(*r* = 22 Å; three layers of ions) around charged nanoparticles with *x* = 22 Å. The large value of *r* = 22 Å was used to compare 2:2 salt with 1:1 salt of bound ions because monovalent ions bind loosely relatively to divalent ions; see Figs 2D–F and 3D–F.

To quantitatively compare the efficiency of monovalent and divalent ions in screening the Coulomb attraction between oppositely charged nanoparticles, we calculated the equivalent 1:1 salt concentrations, which can cause (nearly) the same PMFs as those at 0.1 mM, 1 mM, and 10 mM 2:2 salt concentrations, respectively. Practically, the interpolation technique was used to obtain this equivalent 1:1 salt because the 1:1 concentrations cannot be continuously covered in our MC simulations, and we used ∆*G*(*x* = 22 Å) to compare the PMFs for 1:1 and 2:2 salts because the similar ∆*G*(22 Å) values generally correspond to the similar ∆*G*(*x*) values over separation *x*. It is shown that ∆*G*(*x*) values from 2:2 salts and the respectively equivalent 1:1 salts deduced from ∆*G*(22 Å) values are almost the same over the whole range of separation *x*; see Fig. S5 in the SM. As shown in Fig. 4A, divalent ions are much more efficient than monovalent ions, and this higher efficiency of divalent ions over monovalent ions becomes more pronounced for nanoparticles with higher charge density. For example, 0.1 mM and 10 mM 2:2 salts can cause (nearly) the same attractive PMFs as ~3 mM and ~80 mM 1:1 salts for nanoparticles with |Z| = 9e, respectively. However, for nanoparticles with |Z| = 24e, 0.1 mM and 10 mM 2:2 salts can cause the same PMFs as ~30 mM and ~300 mM 1:1 salts, respectively.

To understand the efficient role of divalent ions over monovalent ions in screening the Coulomb attraction between oppositely charged nanoparticles, we further calculated another kind of equivalent 1:1 salt concentration to 2:2 salt by comparing the net ion charge fraction *Q*(*r*); see Eq. 1. Practically, we also used the interpolation technique and *Q*(*r* = 22 Å; three layers of ions) with the separation *x* = 22 Å to compare 1:1 salts and 2:2 salts. Here, the large value of *r* = 22 Å was used to compare 2:2 salt with 1:1 salt of bound ions because monovalent ions bind loosely relative to divalent ions. As shown in Fig. 4B, the relationship between the equivalent 1:1 salt and 2:2 salts from *Q*(*r* = 22 Å) shares a similar trend to that from ∆*G*(*x* = 22 Å) in Fig. 4A; i.e., to achieve the same ionic neutralization, divalent ions are much more efficient than monovalent ions, and this apparently higher efficiency of divalent ions becomes more pronounced for nanoparticles with higher charge density. Therefore, the comparison between Fig. 4A and B clearly shows that the more efficient role of divalent ions than monovalent ions in screening Coulomb attraction between oppositely charged nanoparticles is attributed to the larger charge fraction of bound divalent ions.

### Deviation between the PB theory and MC simulations and an apparent parameter

**Figure 5:**
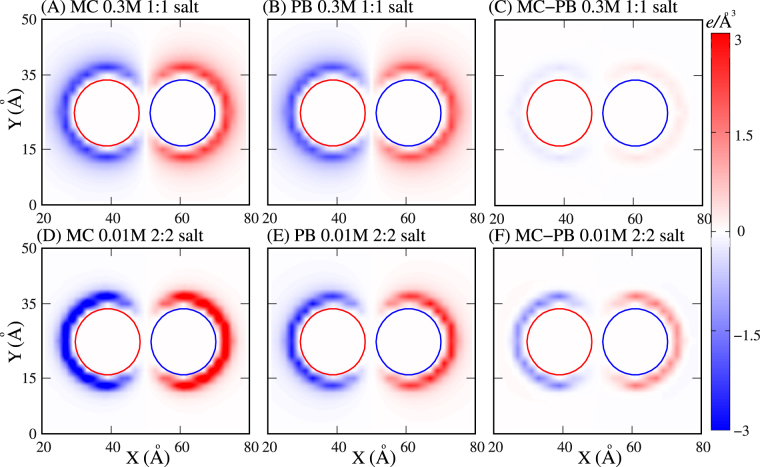
(**A**,**B**) Distribution landscapes of net ion charge density around two oppositely charged nanoparticles with |Z| = 18e and *x* = 22 Å in 0.3 M 1:1 salt solution from the MC simulations (**A**) and the PB theory (**B**). (**C**) Deviation between the distributions of net ion charge density calculated by the PB theory and the MC simulations. (**D**,**E**) Distribution landscapes of net ion charge density around two oppositely charged nanoparticles with |Z| = 18e and *x* = 22 Å in 0.01 M 2:2 salt solution from the MC simulations (**D**) and the PB theory (**E**). (**F**) Deviation between the distributions of ion net charge density calculated by the PB theory and MC simulations. The circles represent the surfaces of nanoparticles.

To obtain a direct understanding on the deviation between the PMFs from the PB theory and MC simulations, we further calculated the charge density distribution landscapes that include both cations and anions around the nanoparticles with |Z| = 18e and *x* = 22 Å. As shown in Fig. 5, the distributions of net ion charge density around nanoparticles from the PB theory and MC simulations share similar landscapes. For high 1:1 salt concentration, the deviation of ion net charge density appears slight, whereas such deviation becomes pronounced for high 2:2 salt concentration.

**Figure 6:**
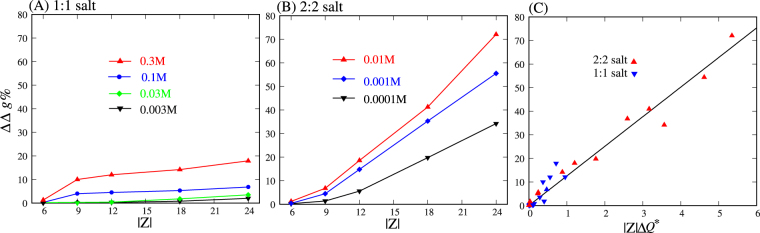
Relative deviation ∆∆*g* (Eq. 2) between the PMFs calculated by the PB theory and MC simulations for two oppositely charged nanoparticles with separation of *x* = 22 Å in 1:1 (**A**) and 2:2 (**B**) salt solutions. (**C**) The relative deviation ∆∆*g* as a function of the apparent parameter |Z|∆Q* between the PB theory and MC simulations, which was defined as |Z|∆*Q*(*r*) at *r* = 14 Å, where the maximum values of |Z|∆*Q*(*r*) are located at high 1:1 and 2:2 salts; see Figs 2 and 3. Here, *x* = 22 Å was used because the deviation between PMFs from the PB theory and MC simulations is most apparent over the covered separation range.

In the following, we quantitatively examine the relative deviation of the PMFs between the PB theory and the MC simulations, which is characterized by

2 $${\rm{\Delta }}{\rm{\Delta }}g=|\frac{{\rm{\Delta }}{G}_{{\rm{MC}}}({x}_{0})-{\rm{\Delta }}{G}_{{\rm{PB}}}({x}_{0})}{{\rm{\Delta }}{G}_{{\rm{MC}}}({x}_{0})}|$$

here, x_0_ = 22 Å was used because the maximum deviation of PMFs between the PB theory and the MC simulations are generally located at x_0_ = 22 Å; see Figs S2A–C and S4A–C in the SM. As shown in Fig. 6A,B, the relative difference ∆∆*g* between the PMFs from the PB theory and the MC simulations is generally rather small at low 1:1 salt concentrations and becomes visible at high 1:1 salt concentrations. For example, for |Z| = 24e, ∆∆*g* is ~3.5% in 0.03 M 1:1 salt and can reach ~15% in 0.3 M 1:1 salt. In contrast, for 2:2 salt, the relative deviation ∆∆*g* generally appears significant except for nanoparticles with very low |Z| and becomes more pronounced for nanoparticles with higher |Z| and for higher 2:2 salt concentrations. For example, in 0.1 mM 2:2 salt solution, ∆∆*g* is ≤~7% for the nanoparticles with |Z| ≤ 12e and becomes ~34% for those with |Z| = 24e. When the 2:2 salt concentration is increased to 10 mM, ∆∆*g* becomes as large as ~18% for the nanoparticles with |Z| = 12e and ~73% for those with |Z| = 24e. Therefore, for two oppositely charged nanoparticles in 1:1 salt solution, the PB theory generally predicts the PMFs between them reliably except for very high salt concentration (~0.3 M). However, for oppositely charged nanoparticles in 2:2 salt solution, the PB theory generally apparently overestimates the attractive PMFs except for the nanoparticles with very low |Z|.

To further characterize the relative deviation ∆∆*g* of the PMFs between the PB theory and MC simulations, an apparent parameter |Z|∆*Q* ^*^ of the difference in total net ion charges was defined as |Z|∆*Q*(*r*) at *r* = 14 Å, where the maximum values of |Z|∆*Q*(*r*) are located at high 1:1 and 2:2 salts; see Figs S2D–F and S4D–F in the SM. Figure 6C showed the relative difference ∆∆*g* as a function of |Z|∆Q* for both 1:1 and 2:2 salts. It is interesting that ∆∆*g* appears tightly and positively coupled to |Z|∆*Q* ^*^ over wide ranges of salt conditions and charge densities on nanoparticles. This tight and positive coupling between ∆∆*g* and |Z|∆*Q* ^*^ is reasonable. Because bound ions mainly provide ionic neutralization to reduce the Coulomb attraction between oppositely charged nanoparticles, the large difference in bound ion charges should correspond to the large deviation in the PMFs between the PB theory and MC simulations. Therefore, |Z|∆*Q* ^*^ can be served as an apparent parameter to well characterize the deviation degree of the PMFs between the PB theory and MC simulations for oppositely charged nanoparticles.

## Conclusions

In this work, both the PB theory and MC simulations were employed to investigate the PMFs between oppositely charged nanoparticles in 1:1 and 2:2 salt solutions. The work covers wide ranges of 1:1 and 2:2 salt concentrations and charge densities on nanoparticles. Through systematic calculations and detailed analyses, the following conclusions have been obtained:

1. For 1:1 salt solutions, the PMFs between oppositely charged nanoparticles are always attractive and become more attractive for nanoparticles with higher charge density and in lower 1:1 salt solution. The PMFs from the PB theory are generally in good agreement with those from the MC simulations except for the slight deviation at very high 1:1 salt concentrations, which suggests that the PB theory can generally describe the PMFs between oppositely charged nanoparticles in 1:1 salt solutions.
2. For 2:2 salt solutions, the PMFs between oppositely charged nanoparticles are always attractive, and this effective attraction becomes stronger for nanoparticles with higher charge density and lower 2:2 salt concentration. The PB theory generally overestimates the attractive PMFs between oppositely charged nanoparticles in 2:2 salt solutions, especially for highly charged nanoparticles and for high 2:2 salt concentrations.
3. Compared with 1:1 salt, 2:2 salt is more efficient in screening the Coulomb attraction between oppositely charged nanoparticles, and this efficient role of 2:2 salt solutions is more pronounced for more highly charged nanoparticles.
4. The overestimation of the PB theory on the attractive PMFs is attributed to the underestimation of bound ions around charged nanoparticles. The relative deviation in the PMFs is tightly and positively coupled to an apparent parameter |Z|∆Q* of the difference in the total net ion charges between the PB theory and MC simulation for oppositely charged nanoparticles.

Our conclusion that the PB theory generally overestimates the attractive PMFs between oppositely charged nanoparticles in 2:2 salt solutions is attributed to the higher ionic charge and the resultant strong ion correlations, as discussed above. This is not contradictory to the combination work of atomic force microscopy and the PB equation with charge regulation, which can be attributed to the change of effective charges on nanoparticles due to possible ion binding or releasing in the inner layer of nanoparticles^68–70^. The present work was mainly focused on the nanoparticles with constant charges, and our PB calculations based on the original version of the PB theory did not involve charge regulation in the inner layers of nanoparticles. Our conclusions do not exclude the validity of the PB equation with charge regulation on the PMFs between oppositely charged nanoparticles mediated by multivalent ions, especially for low salt concentrations and at large separations^68–70^, because the effective charges on nanoparticles can be apparently reduced by the ion binding in the inner layers of nanoparticles and consequently the electrostatic potential in the diffusive layers can be greatly reduced^68–70^. Moreover, in the present work, the PMFs between oppositely charged nanoparticles are calculated as the effective attractions in 1:1 and 2:2 salt solutions, which is also not contradictory to the effective repulsions observed in previous experimental and theoretical studies^42,43,71–76^. The effective repulsions in previous studies might be attributed to the adopted very high asymmetrical salts and the resultant over-neutralization of bound multivalent ions to the nanoparticles^42,43,73,75,76^. However, the present work involves only symmetrical 1:1 and 2:2 salts and does not cover very high salt concentrations, thus only predicts effective attractions between oppositely charged nanoparticles.

The present work also involves some approximations and simplifications. First, the solvent molecules were modelled implicitly as a continuum medium with a high dielectric constant of 78, and for simplicity, the dielectric discontinuity at the interface between nanoparticles and solvent was ignored^35,50,80,81^. This assumption may slightly affect the bound ions at the nanoparticle surface and should be considered in future work. Second, the nanoparticles and ions were treated as hard-core spheres, which should not essentially affect our results and analyses. Finally, the present work did not cover the effective interactions at very large separation (e.g., ≫ nanoparticle size), because the assembly of nanoparticles generally involves interactions within a moderate separation range^68–70^. Nevertheless, the present work illustrates that the effective attraction between oppositely charged nanoparticles can be greatly modulated by salt ions, especially by multivalent ions. In addition, this work suggests that the PB theory can generally provide reliable predictions on the effective interactions between oppositely charged nanoparticles in 1:1 salt solutions, while it cannot quantitatively describe the PMFs between oppositely charged nanoparticles in multivalent salt solutions. The present work would be very helpful for understanding the ion-mediated assembly and complexation of oppositely charged polyelectrolytes^42–45,71–76^.

## Model and Method

### Model system for oppositely charged nanoparticles

In this work, for simplicity, we employed a model system of two oppositely charged nanoparticles immersed in 1:1 and 2:2 salt solutions to study the PMFs between oppositely charged nanoparticles^50–52^. In the model system, both charged nanoparticles and salt ions were represented by spheres with radii of 10 Å and 2 Å, and the solvent was modelled as a continuous medium with dielectric constant *ε* = 78; see Fig. 1. The charges on two oppositely charged nanoparticles were taken as +6e/−6e, +9e/−9e, +12e/−12e, +18e/−18e and +24e/−24e. In the model system, the interactions between nanoparticles and ions were simplified into Coulomb and hard-sphere repulsion interactions, and the interaction energy *U*_*ij*_ between spheres *i* and *j* in the model system is given by

3 $${U}_{ij}=\{\begin{array}{c}\frac{{q}_{i}{q}_{j}}{4\pi {\varepsilon }_{0}\varepsilon {r}_{ij}},\,{{\rm{r}}}_{{\rm{ij}}}\ge {\sigma }_{i}+{\sigma }_{j};\\ +\infty ,\,{{\rm{r}}}_{{\rm{ij}}} < {\sigma }_{i}+{\sigma }_{j}.\end{array}$$

Here, *σ*_*i*_ and *q*_*i*_ represent the radius and charge of particle *i* (nanoparticles or ions). *r*_*ij*_ is the distance between the centres of two charged particles *i* and *j* (nanoparticles and ions). *ε* is the dielectric constant of solvent, and *ε*_0_ is the permittivity of vacuum. Thus, our systems involved only the effects of Coulomb and exclusion volume interactions and did not involve possible chemical processes such as specific proton adsorption or surface ionization in some colloid systems^68–70^.

### Monte Carlo simulations for PMFs

For the model system, the MC simulations with the Metropolis algorithm have been employed to calculate the PMFs between oppositely charged nanoparticles in salt solutions. The simulation cell was generally a rectangular box, and to diminish the boundary effect, the box size was kept larger than the centre-to-centre separation *x* between oppositely charged nanoparticles by at least six Debye–Hückel lengths^35–37,50^.

Following previous studies^46,49,53^, we employed the pseudo-spring method to calculate PMFs^46,49,52^, and a spring with spring constant *k* = 9 nN/Å was added to link the centres of the two oppositely charged nanoparticles. In our MC simulations, one nanoparticle remains frozen, while the other can move along x-axis with a constraint of the added spring. The effective force *F*(*x*) between the two nanoparticles with a centre-to-centre separation *x* can be given by^46,49,53^

4 $$F(x)=k{\rm{\Delta }}x,$$

where ∆*x* is the small deviation of spring length away from the original distance *x* at equilibrium. The convergence of the average separation between two nanoparticles is shown in Fig. S6 of the SM. Afterwards, the PMF ∆*G*(*x*) between the two nanoparticles can be calculated by the following integration^46,49,53^:

5 $${\rm{\Delta }}G(x)={G}({x})-{G}({{x}}_{{\rm{ref}}})={\int }_{x}^{{x}_{{\rm{ref}}}}F(x^{\prime} )dx^{\prime} ,$$

where *x*_ref_ is the outer reference distance, which was taken as 40 Å in practice^43,46,50^. More details about the MC simulations can be found in Section 1 of the SM.

### The Poisson–Boltzmann theory for PMFs

In a parallel way, the PB theory has been employed to calculate the PMFs between oppositely charged nanoparticles in 1:1 and 2:2 salt solutions, because the PB theory is a well-established polyelectrolyte theory^24–32^. The electrostatic potential *ψ*(**r**) around charged nanoparticles can be given by the PB equation^24,26,29,35,37,50^

6 $$\nabla \cdot [{\varepsilon }_{0}\varepsilon \nabla \psi ({\bf{r}})]=-4{\pi }\{{\rho }_{{\rm{f}}}+\sum _{i}{z}_{i}e{c}_{i}^{0}{e}^{-\beta {z}_{i}e\psi ({\bf{r}})}\},$$

where *ρ*_f_ is the charge density of fixed charges on nanoparticles, *z*_*i*_ is the valance of ion species *i*, and ${c}_{i}^{0}$ denotes the bulk concentration of species *i*. *β* = 1/*k*_B_ *T*, where *T* is the absolute temperature in Kelvin, and *k*_B_ is the Boltzmann constant. With electrostatic potential from the PB equation, the electrostatic free energy for two nanoparticles with a centre-to-centre separation *x* can be calculated by^30–32,37,50^

7 $${G}_{{\rm{PB}}}({x})=\frac{1}{2}\int \sum _{i}{c}_{i}({\bf{r}}){z}_{i}e[\psi ({\bf{r}})+{\psi }_{{\rm{f}}}({\bf{r}})]{{\rm{d}}}^{3}{\bf{r}}+{k}_{B}T\int \sum _{i}[{c}_{i}({\bf{r}})\mathrm{ln}\,\frac{{c}_{i}({\bf{r}})}{{c}_{i}^{0}}-{c}_{i}({\bf{r}})+{c}_{i}^{0}]{{\rm{d}}}^{3}{\bf{r}}+{U}_{{\rm{N}}-{\rm{N}}},$$

where *U*_N-N_ is the Coulomb energy for the oppositely charged nanoparticles. $\psi ({\bf{r}})$ and ${\psi }_{f}({\bf{r}})$ are the electrostatic potentials with and without diffusive salt ions, and ${c}_{i}({\bf{r}})={c}_{i}^{0}{e}^{-\beta {z}_{i}e\psi ({\bf{r}})}$ denotes the ion concentration of species *i* at **r**. It is noted that the electrostatic potential $\psi ({\bf{r}})$ changes with separation *x* between two nanoparticles. The derivation of Eq. 7 is detailed in the SM; see Section 2 in the SM. Therefore, ${\rm{\Delta }}{{G}}_{{\rm{PB}}}({x})={G}_{{\rm{PB}}}({x})-{G}_{{\rm{PB}}}({x}_{{\rm{ref}}})$ gives the PMF between two nanoparticles by the PB theory, where *x*_ref_ is taken as 40 Å^35–37,46,49,50,53^. Thus, our PB calculations are based on the original version of the PB theory, without considering charge regulation due to ion binding or releasing in the inner layers of nanoparticles^68–70^.

In this work, the three-dimensional algorithm developed in the tightly bound ion theory was used to numerically solve the PB equation^35–37,50^. To account for the excluded volume layer of ions, a thin layer of ion radius was added to the nanoparticle surface, and the charge density *ρ*_f_ of fixed charges on nanoparticles can be obtained approximately by partitioning the fixed charges to their eight nearest grid points when solving the PB equation on a cubic lattice with the finite-difference method^35–37,50^. The focusing process of three steps has been used to compute the detailed electrostatic potential near the nanoparticle surface^35–37,50^. In the first step, the PB equation was solved numerically on a cubic box with large grid size, which depends on salt concentration. Generally, we chose a box-size approximately six times larger than the Debye–Hückel length from the nanoparticle surface to include the salt effect in solution^35–37,50^. In the second step of focusing, the PB was solved on a smaller cubic box with smaller grid size, and the initial electrostatic potentials were obtained from the interpolation based on those in the first step. A similar procedure was repeated in the third focusing step to obtain the electrostatic potentials with high resolution near the nanoparticles. The resolution of the first step varies with the grid size to increase the efficiency of the iterative process. The grid sizes (*L*_x_, *L*_y_, *L*_z_) were kept at (160 Å, 120 Å, 120 Å) and (100 Å, 60 Å, 60 Å) in the second and the third focusing steps, respectively, and the corresponding resolutions were 1.0 Å and 0.25 Å per grid size. As a result, the numbers of grid points were 161 × 121 × 121 in the second step and 401 × 241 × 241 in the third step. The iteration for each step was continued until the electrostatic potential change *δψ*(**r**) for an iteration was less than 10^−4^ *k*_B_ *T*/*e* ^35–37,50^.

## Electronic supplementary material

Supplementary Material
