# Supplementary material for: Potential of mean force between oppositely charged nanoparticles: A comprehensive comparison between Poisson–Boltzmann theory and Monte Carlo simulations
Source: Sci Rep. 2017 Oct 26;7:14145. doi: 10.1038/s41598-017-14636-x (PMC5658377; doi:10.1038/s41598-017-14636-x)
Supplement: Supplementary file 1 — Supplementary Material [file 41598_2017_14636_MOESM1_ESM.doc]

Supplementary Material for

**Potential of mean force between oppositely charged nanoparticles: A comprehensive comparison between Poisson–Boltzmann theory and Monte Carlo simulations**

Jin-Si Zhang^†^, Xi Zhang^†^, Zhong-Liang Zhang, and Zhi-Jie Tan^*^

*Center for Theoretical Physics and Key Laboratory of Artificial Micro & Nano-structures of Ministry of Education, School of Physics and Technology, Wuhan University, Wuhan 430072, China*

^†^The authors contributed equally to the work.

^*^To whom correspondence should be addressed: zjtan@whu.edu.cn

**1. Details of the Monte Carlo simulations for calculating potentials of mean force**

As described in the main text, we employed Monte Carlo (MC) simulations with the pseudo-spring method to calculate the potential of mean force between two oppositely charged nanoparticles. In our MC simulations, the pseudo-spring with spring constant *k* = 9 nN/Å is added to link the centres of two oppositely charged nanoparticles along the x-axis. The simulation box sizes and ion numbers are shown in Table S1 of the Supplementary Material. During the MC simulations, one nanoparticle remains frozen, while the other can move along x-axis with a small deviation Δ*x* from the original separation *x* between two nanoparticles due to the constraint of the spring. The steps of the trial movement are 0.5 Å for ions and a random value in the range of [0, 0.1 Å] for the mobile nanoparticle. The energy change of the system due to a trial movement of ions or the mobile nanoparticle is calculated, and the acceptance probability is determined by the Metropolis algorithm. For the different cases we covered, the average acceptance probabilities of ions and the mobile nanoparticle are 60–80% and 65–75%, respectively. After Δ*x* reaches a convergence (generally before ~3×10^7^ MC steps), more than ~8×10^7^ steps were generally used for calculating the average force; see Table S1 and Fig. S6 in the Supplementary Material. Specifically, we chose eight separations *x* (22 Å, 24 Å, 26 Å, 28 Å, 30 Å, 33 Å, 36 Å and 40 Å) to calculate the potentials of mean force, where the second-order integration of the composite Simpson’s rule for unequally spaced ordinates [1,2] was employed because the average force *F*(*x*) is a concave function of the centre-to-centre separation *x* between nanoparticles. We also calculated the potentials of mean force using the composite trapezoidal rule. The differences between the potentials of mean force from the composite trapezoidal rule and the composite Simpson’s rule for unequally spaced ordinates are found to be small; see Fig. S7 in the Supplementary Material.

Table S1. Box sizes, ion numbers and numbers of MC steps in our MC simulations

| salt conditions | box sizes (Å^3^) | ion numbers^a)^ | total number of MC steps |
| --- | --- | --- | --- |
| 0.3M 1:1 salt | 130×90×90 | 380 | 1.8×10^8^ |
| 0.1M 1:1 salt | 160×120×120 | 276 | 1.2×10^8^ |
| 0.03M 1:1 salt | 250×150×150 | 202 | 2.5×10^8^ |
| 0.003M 1:1 salt | 350×350×350 | 154 | 2.3×10^8^ |
| 0.01M 2:2 salt | 250×250×250 | 188 | 1.1×10^8^ |
| 0.001M 2:2 salt | 600×600×600 | 260 | 2.6×10^8^ |
| 0.0001M 2:2 salt | 1200 1200×1200×1200 | 208 | 3.0×10^8^ |

^a)^ The total numbers of cations and anions in the respective simulation box.

**2. Derivation of Eq. 7**

For two charged nanoparticles with center-to-center separation *x* in a salt solution, the free energy *G*_PB_ of the PB theory is composed of two contributions *G*_entropy_ and *G*_el_

$G_{PB}(x)=G_{entropy}(x)+G_{el}(x).$ (S1)

The first term *G*_entropy_ is the free energy of entropy caused by the reorganization of ions due to the existence of charged nanoparticles, and can be given by [3-9]

$G_{entropy}(x)=k_{B}T\int\sum_{i} \left[c_{i}(r)ln\frac{c_{i}(r)}{c_{i}^{0}}-c_{i}(r)+c_{i}^{0}\right]d^{3}r,$ (S2)

where $c_{i}^{0}$ and $c_{i}(r)$ denote bulk ion concentration and ion concentration at **r** of ion species *i*, respectively. The second term *G*_el_ of Eq. S1 is the electrostatic energy of the system, which is given by [6-10]

$G_{el}(x)=\frac{1}{2}\int\psi(r)\rho(r)d^{3}r=\frac{1}{2}\int\left[\psi_{f}(r)+\psi_{d}(r)\right]\cdot\left[\rho_{f}(r)+\rho_{d}(r)\right]d^{3}r,$ (S3)

where $\psi(r)$ ($=\psi_{f}(r)+\psi_{d}(r)$) and $\rho(r)$ ($=\rho_{f}(r)+\rho_{d}(r)$) denote the electrostatic potential and charge density at **r**, and 1/2 is used to avoid double-counting. $\psi_{f}(r)$ and $\psi_{d}(r)$ represent the contributions of fixed charges and diffusive ions to $\psi(r)$, respectively. $\rho_{f}(r)$ and $\rho_{d}(r)$ represent the charge densities of fixed charges (charged nanoparticles) and diffusive ions, respectively. Thus, Eq. S3 can be further written as

$G_{el}(x)=\frac{1}{2}\int\psi_{f}(r)\rho_{f}(r)d^{3}r+\frac{1}{2}\int\left[\psi_{f}(r)\rho_{d}(r)+\psi_{d}(r)\rho_{f}(r)\right]d^{3}r+\frac{1}{2}\int\psi_{d}(r)\rho_{d}(r)d^{3}r$ (S4)

which is also consistent with previous references [6,7]. The first term in the right hand of Eq. S4 is the Coulomb energy *U*_N-N_ for the oppositely charged nanoparticles

$U_{N-N}=\frac{1}{2}\int\psi_{f}(r)\rho_{f}(r)d^{3}r.$ (S5)

Furthermore, because the Coulomb interactions between fixed charges (charged nanoparticles) and diffusive ions should be equal, i.e.,$\int\psi_{f}(r)\rho_{d}(r)d^{3}r=\int\psi_{d}(r)\rho_{f}(r)d^{3}r$, Eq. S4 can be changed into

$G_{el}(x)=U_{N-N}+\int\psi_{f}(r)\rho_{d}(r)d^{3}r+\frac{1}{2}\int\psi_{d}(r)\rho_{d}(r)d^{3}r.$ (S6)

Here, we chose the expression without *ρ*_f_, in order to reduce the sensitivity of grid-dependence in numerical integration for calculating *G*_el_ [5]. Since the contribution of diffusive ions $\psi_{d}(r)$ to the electrostatic potential $\psi(r)$ is given by $\psi_{d}(r)=\psi(r)-\psi_{f}(r)$, Eq. S6 can be further changed into

$\begin{matrix} G_{el}(x)=U_{N-N}+\int\psi_{f}(r)\rho_{d}(r)d^{3}r+\frac{1}{2}\int\left[\psi(r)-\psi_{f}(r)\right]\rho_{d}(r)d^{3}r \\ \text{ }=U_{N-N}+\frac{1}{2}\int\left[\psi(r)+\psi_{f}(r)\right]\rho_{d}(r)d^{3}r. \end{matrix}$ (S7)

In Eq. S7, the charge density$\rho_{d}(r)$of diffusive ions is given by$\rho_{d}(r)=\sum_{i} c_{i}(r)z_{i}e$, where *z_i_* is the valence of ion species *i*. Therefore, based on Eqs. S2 and S7, the free energy of the PB theory for two charged nanoparticles in a salt solution has the following final form

$G_{PB}(x)=\frac{1}{2}\int\sum_{i} c_{i}(r)z_{i}e\left[\psi(r)+\psi_{f}(r)\right]d^{3}r+k_{B}T\int\sum_{i} \left[c_{i}(r)ln\frac{c_{i}(r)}{c_{i}^{0}}-c_{i}(r)+c_{i}^{0}\right]d^{3}r+U_{N-N}$. (S8)

This is just Eq. 7 in the main text. Through solving the PB equation, we can obtain $\psi(r)$ and $\psi_{f}(r)$, the electrostatic potentials with and without diffusive salt ions. Afterwards, we can calculate *G*_PB_ through Eq. 7 or Eq. S8.

**Figure S1** (A-B) Potentials of mean force Δ*G* as functions of separation *x* between the centers of two oppositely charged nanoparticles in 1:1 salt solutions, which were calculated by the PB theory and MC simulations respectively. The insets show the deviation between the PMFs calculated by the PB theory and the MC simulations: ∆∆*G*(*x*) = ∆*G*_MC_(*x*) – ∆*G*_PB_(*x*). (C-D) Net charge distributions *Q*(*r*) per unit charge on nanoparticles as functions of distance *r* around the nanoparticles with *x* = 22 Å in 1:1 salt solutions; see Eq. 1. The error bars are shown in the figure.

**Figure S2** (A-C) Deviation ΔΔ*G*(*x*) between the PMFs calculated by the PB theory and the MC simulations: ∆∆*G*(*x*) = ∆*G*_MC_(*x*) – ∆*G*_PB_(*x*) with separation *x* in 1:1 salt solutions. (D-F) Deviation |Z|∆*Q*(*r*) between the total net charges |Z|*Q*(*r*) calculated by the PB theory and the MC simulations: |Z|∆*Q*(*r*) = |Z|(*Q*_MC_(*r*) – *Q*_PB_(*r*)) with a distance *r* from the center of the nanoparticle in 1:1 salt solutions.

**Figure S3** (A-B) Potentials of mean force Δ*G* as functions of separation *x* between the centers of two oppositely charged nanoparticles in 2:2 salt solutions, which were calculated by the PB theory and MC simulations respectively. The insets show the deviation between the PMFs calculated by the PB theory and the MC simulations: ∆∆*G*(*x*) = ∆*G*_MC_(*x*) – ∆*G*_PB_(*x*). (C-D) Net charge distributions *Q*(*r*) per unit charge on nanoparticles as functions of distance *r* around the nanoparticles with *x* = 22 Å in 2:2 salt solutions; see Eq. 1. The error bars are shown in the figure.

**Figure S4** (A-C) Deviation ΔΔ*G*(*x*) between the PMFs calculated by the PB theory and the MC simulations: ∆∆*G*(*x*) = ∆*G*_MC_(*x*) – ∆*G*_PB_(*x*) with separation *x* in 2:2 salt solutions. (D-F) Deviation |Z|∆*Q*(*r*) between the total net charges |Z|*Q*(*r*) calculated by the PB theory and the MC simulations: |Z|∆*Q*(*r*) = |Z|(*Q*_MC_(*r*) – *Q*_PB_(*r*)) with a distance *r* from the center of the nanoparticle in 2:2 salt solutions.

**Figure S5** (A-E) Potentials of mean force Δ*G* as functions of separation *x* between the centers of two oppositely charged nanoparticles in 2:2 salt solutions (dashed lines), and in the corresponding equivalent 1:1 salt solutions (solid lines) obtained through comparing ∆*G*(*x* = 22 Å). Here, ∆*G*(*x*)’s for equivalent 1:1 salts are obtained from the interpolations as described in the main text.

**Figure S6** Convergences of the average center-to-center separation between two nanoparticles in 0.3 M 1:1 solution (A) and 0.01 M 2:2 solution (B) along Monte Carlo steps. The spring lengths *x*_0_ of the pseudo-spring method are 22 Å in the upper panels and 40 Å in the bottom panels.

**Figure S7** (A-D) Potentials of mean force Δ*G* calculated using the composite Simpson’s rule for unequally spaced ordinates (solid lines) and the composite trapezoidal rule (dashed lines) in 1:1 (A-B) and 2:2 (C-D) salt solutions.

**Reference**

1. Shklov, N. Simpson's rule for unequally spaced ordinates. *American Mathematical Monthly,* 67, 1022-1023 (1960).
2. Press, W. H. *Numerical recipes 3rd edition: The art of scientific computing*. Cambridge university press (2007).
3. Theodoor, J., & Overbeek, G. The role of energy and entropy in the electrical double layer. *Colloids and Surfaces* **51**, 61-75(1990).
4. Stigter, D. Evaluation of the counterion condensation theory of polyelectrolytes. *Biophys. J.* **69**, 380-388 (1995).
5. Tan, Z. J., & Chen, S. J. Electrostatic correlations and fluctuations for ion binding to a finite length polyelectrolyte. *J. Chem. Phys.* **122**, 044903 (2005).
6. Grochowski, P., & Trylska, J. Continuum molecular electrostatics, salt effects, and counterion binding—a review of the Poisson–Boltzmann theory and its modifications.*Biopolymers* **89**, 93-113. (2008).
7. Markovich, T., Andelman, D., & Podgornik, R. Charged Membranes: Poisson-Boltzmann theory, DLVO paradigm and beyond. *arXiv preprint arXiv*:1603, 09451 (2016).
8. Korolev, N., Lyubartsev, A. P., & Nordenskiöld, L. Application of polyelectrolyte theories for analysis of DNA melting in the presence of Na^+^ and Mg^2+^ ions. *Biophys. J.* **75**, 3041-3056 (1998).
9. Korolev, N., Lyubartsev, A. P., & Nordenskiöld, L. Application of the Poisson Boltzmann polyelectrolyte model for analysis of equilibria between single-, double-, and triple-stranded polynucleotides in the presence of K^+^, Na^+^, and Mg^2+^ ions. *J. Biomol. Struct. Dyn.* **20**, 275-290 (2002).
10. Jackson, J. D. Classical electrodynamics. John Wiley & Sons (2007).
